# Numerical Simulation of Thermal Processes in a Domain of Thin Metal Film Subjected to an Ultrashort Laser Pulse

**DOI:** 10.3390/ma11112116

**Published:** 2018-10-28

**Authors:** Ewa Majchrzak, Bohdan Mochnacki

**Affiliations:** 1 Faculty of Mechanical Engineering, Silesian University of Technology, 44-100 Gliwice, Poland; 2 Department of Technical Sciences, University of Occupational Safety Management in Katowice, 40-007 Katowice, Poland; bmochnacki@wszop.edu.pl

**Keywords:** microscale heat transfer, dual phase lag equation, melting and resolidification, numerical modeling, finite difference method

## Abstract

A thin metal film subjected to an ultrashort laser pulse is considered. With a sufficiently high laser intensity the process of the film heating may cause metal melting and even ablation. In this work, the numerical model of the melting and resolidification processes is presented. The mathematical model is based on the dual phase lag equation in which two positive constants appear, this means the relaxation and thermalization times. The considered equation contains a second-order time derivative and higher order mixed derivative in both time and space and should be supplemented by the appropriate boundary and initial conditions. The model of the melting and resolidification is presented in two versions. The first can be called ‘the introduction of the artificial mushy zone sub-domain’, while the second ‘the two forms of the basic energy equation’. At the stage of numerical computations, the implicit scheme of the finite difference method is used. The numerical algorithm is tested for the two proposed models which are applied to the computations concerning the thermal processes occurring in the cylindrical micro-domain (chromium, gold) subjected to an ultrashort laser pulse.

## 1. Introduction

In this paper, the application of the dual-phase lag equation (DPLE—see: Section 2) [1,2,3,4] for numerical modeling of the problems related to the microscale heat transfer are discussed. In particular, a thin metal film subjected to a laser pulse is analyzed. Generally speaking, the differences between the macroscopic heat conduction equation basing on the Fourier law and the models describing the ultrafast laser pulse interactions with the metal films appear because of an extremely short duration, extreme temperature gradients and very small dimensions of considered domain [5]. The delay times which are irrelevant in the case of the macroscopic heat transfer in the metals, play an important role in the analyzed case. As a rule, the DPLE of the first-order is considered (this is determined by the number of components in the Taylor series development), but the other forms of DPLE are also discussed. For example, both the heat flux **q** and the temperature gradient ∇*T* in the generalized Fourier law are expanded using the second-order Taylor formula (a second-order DPLE) [6,7]. The numerical solution of the second-order DPLE using the implicit scheme of the finite difference method (FDM) is reported by Chiriţă [8]. The mixed variants of DPLE are also considered, e.g., the second-order Taylor expression of heat flux and the first-order Taylor expression of temperature gradient [9]. The physical correctness of the higher order DPLE solutions and the limitations concerning the lag times are discussed by Fabrizio and Quintanilla [10,11].

A majority of the analytical or semi-analytical solutions of equations with two delay times refer to the one-dimensional (1D) tasks, homogeneous domains and simple boundary-initial conditions. An analytical solution devoted to the thermal processes occurring in the plate irradiated by the short pulse laser is presented by Tang and Araki [12]. The solution is obtained using the Green function method and finite integral transform technique. The solution concerning the heating of the plate in which the thermal processes are described by the higher order DPLE supplemented by the Dirichlet or Neumann boundary conditions and initial ones is reported by Castro et al. [13]. The interesting (from the practical point of view) analytical solution is discussed by Ciesielski [14]. The author solves the 1D first-order DPLE describing the heating/cooling process in the thin metal film subjected to a laser pulse. The laser action is taken into account by the introduction of the internal heat source (as in the presented paper). To find the analytical solution of the three-dimensional DPLE, the Adomian decomposition method (ADM) and the Adomian double decomposition method are used by Mohammadi-Fakhar and Momeni-Masuleh [15]. To solve the 1D dual-phase lag equation for the non-homogeneous (multi-layered) cylindrical or spherical domain, the Laplace transform solution method is used by Ramadan [16]. The dual phase lag model has (in recent years) been applied in the scope of bio-heat transfer problems, too. Here, two papers can be mentioned. The analytical solution of the dual phase lag bio-heat transfer equation using the finite integral transform is reported by Kumar and Srivastava [17], while the analysis of thermal damage to the laser irradiated tissue is done by Liu and Wang [18].

A large majority of the problems described by DPLE are solved using the numerical methods. Generally, the different variants of the finite difference method are applied, but the solutions based on the boundary element method [19], the finite element method [20,21,22], the control volume method [23,24,25] or the lattice Boltzmann method [26,27] can be also found.

Wang et al. [28] apply the finite difference method for numerical modeling of double-layered thin film heating. Multi-layered domains are considered by Majchrzak et al., also Majchrzak and Kałuża [29,30]. The solution of 1D problem is obtained using the implicit FDM scheme. A three-dimensional FDM numerical model of the thin metal film heating is reported by Dai and Nassar [31]. The explicit scheme of the FDM for numerical solution of DPLE for axially symmetrical domain is used by Mochnacki and Majchrzak [32]. The stability of this type of algorithm is discussed by Majchrzak and Mochnacki [33]. The unconditional stability of the 1D implicit difference scheme is proved by Majchrzak and Mochnacki [34]. In turn, the numerical solution of two-dimensional DPLE using the alternating directions approach can be found by Ciesielski [35]. The FDM is also used for numerical solutions of inverse problems related to the identification of the lag times or other parameters appearing in DPLE (e.g., [25,36]). It should be emphasized that the number of papers devoted to FDM applications for the numerical solution of the problems described by DPLE is, of course, much bigger.

Depending on the laser intensity and characteristic time of laser pulse in the considered domain, not only the heating and cooling processes are observed but also the process of melting and resolidification can take place. In such a case the basic algorithm should be supplemented with the procedures of phase transformations modeling. It should be pointed out that definitely more phase transformations models are related to the two-temperature approach, e.g., [37,38,39]. A relatively small number of papers is devoted to this problem when the mathematical model is based on the DPLE (e.g., [40,41,42]). In this paper, two models of melting and resolidification using the DPLE are presented. One of them is based on the introduction of the artificial mushy zone sub-domain. In other words, the melting/resolidification at a constant temperature is replaced by the melting/resolidification in the temperature range. The second approach is based on the use of two forms of DPLE. The first form describes the heating and cooling processes in the metal domain and then the internal heat source related to the melting/resolidification process is equal to zero. The second form of DPLE describes the heat transfer processes taking place at the stage of the melting and resolidification. Because of the pure metals, the phase transition proceeds at the constant temperature, and the temperature derivatives with respect to time are equal to zero and it leads to the other form of DPLE.

This paper consists of the following parts. At first, the dual phase lag equation is presented and the analyzed problem is formulated. Next, the DPLE solution based on the implicit scheme of the finite difference method is described and the results of computations are shown. In the final part of the paper, conclusions are formulated. 

## 2. Governing Equations

The start point for the further considerations is a generalized form of the Fourier law [1,2,3,4]

(1) $$q\left(X,t+\tau_{q}\right)=-\lambda \nabla T\left(X,t+\tau_{T}\right),$$

where **q** is a heat flux vector, ∇*T* is a temperature gradient, λ is a thermal conductivity, *X*, *t* denotes the geometrical co-ordinates and time. The positive constants $\tau_{q}$, $\tau_{T}$ correspond to the relaxation time and thermalization time, respectively. The relaxation time $\tau_{q}$ takes into account the small-scale response in time, while the thermalization time $\tau_{T}$ takes into account the small-scale response in space [43].

Using the first-order Taylor series expansions one has

(2) $$q\left(X,t\right)+\tau_{q}\frac{\partial q\left(X,t\right)}{\partial t}=-\lambda \left[\nabla T\left(X,t\right)+\tau_{T}\frac{\partial \nabla \left(X,t\right)}{\partial t}\right].$$

From Equation (2) results

(3) $$-q\left(X,t\right)=\tau_{q}\frac{\partial q\left(X,t\right)}{\partial t}+\lambda \left[\nabla T\left(X,t\right)+\tau_{T}\frac{\partial \nabla \left(X,t\right)}{\partial t}\right].$$

Now, the well-known thermal diffusion equation

(4) $$c\frac{\partial T\left(X,t\right)}{\partial t}=-\nabla \cdot q\left(X,t\right)+Q\left(X,t\right)$$

is used. In this equation *c* is the volumetric specific heat and *Q* (*X*, *t*) is the capacity of internal heat sources.

Introducing Equation (3) to Equation (4) one obtains

(5) $$c\frac{\partial T(X,t)}{\partial t}=\tau_{q}\frac{\partial }{\partial t}\left[\nabla \cdot q(X,t)\right]+\nabla \left[\lambda \nabla T(X,t)\right]+\tau_{T}\nabla \left[\lambda \frac{\partial \nabla T(X,t)}{\partial t}\right]+Q(X,t).$$

Because (c.f. Equation (4))

(6) $$\nabla \cdot q\left(X,t\right)=-c\frac{\partial T\left(X,t\right)}{\partial t}+Q\left(X,t\right),$$

thus, in the end [1,2]

(7) $$\begin{array}{l} c\left[\frac{\partial T\left(X,t\right)}{\partial t}+\tau_{q}\frac{\partial^{2}T\left(X,t\right)}{\partial t^{2}}\right]=\nabla \left[\lambda \nabla T\left(X,t\right)\right]+\\ \tau_{T}\frac{\partial }{\partial t}\left\{\nabla \left[\lambda \nabla T\left(X,t\right)\right]\right\}+Q\left(X,t\right)+\tau_{q}\frac{\partial Q\left(X,t\right)}{\partial t} \end{array}.$$

**Figure 1:**
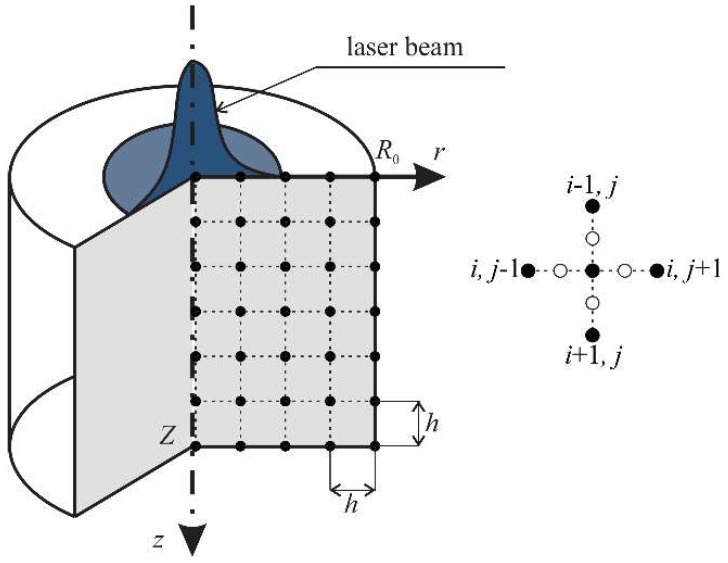
Differential mesh.

The capacity of internal heat source *Q*(*X*, *t*) = *Q*(*r*, *z*, *t*) (the axisymmetric problem is analyzed, as shown in Figure 1) in the considered case is a sum of two components. The first component *Q_L_*(*r*, *z*, *t*) results from the laser action and according to the literature (e.g., [44]) the equation determining this value is the following

(8) $$Q_{L}(r,z,t)=\sqrt{\frac{4\ln2}{\pi }}(1-R)\frac{I_{0}}{\delta t_{p}}\exp\left[-\frac{r^{2}}{r_{D}^{2}}-\frac{z}{\delta }-4\ln2\frac{{\left(t-2t_{p}\right)}^{2}}{t_{p}^{2}}\right],$$

where *I*_0_ [J/m^2^] is a laser intensity, $t_{p}$ [s] is a characteristic time of laser pulse, δ [m] is an optical penetration depth, *R* is a reflectivity of irradiated surface, $r_{D}$ [m] is a laser beam radius. The derivative of *Q_L_* with respect to time can be found analytically. The second component *Q_ph_*(*r*, *z*, *t*) complementary the source function *Q*(*r*, *z*, *t*) is associated with the phase transformations and will be described in the next section.

## 3. One-Domain Approach

The internal heat source resulting from the phase change is proportional to the local and temporary melting/solidification rate, in particular [45,46]

(9) $$Q_{ph}(r,z,t)=L\frac{\partial f_{S}(r,z,t)}{\partial t}=-L\frac{\partial f_{L}(r,z,t)}{\partial t},$$

where *L* is a volumetric latent heat, *f_S_* is a volumetric solid-state fraction in the neighborhood of the point considered, *f_L_* = 1 − *f_S_*. The function *f_L_* is a temperature dependent and for the border temperatures limiting the mushy zone sub-domain takes the values *f_L_* (*T*_1_) = 0 and *f_L_* (*T*_2_) = 1 (*T*_1_ and *T*_2_ correspond to the beginning and the end of the melting process). Thus

(10) $$Q_{ph}(r,z,t)=-L\frac{\partial f_{L}(r,z,t)}{\partial t}=-L\frac{df_{L}(T)}{dT}\frac{\partial T(r,z,t)}{\partial t},$$

where *L* is a volumetric latent heat. Finally, the source term controlling the evolution of latent heat can be written in the form

(11) $$\begin{array}{l} Q_{ph}(r,z,t)+\tau_{q}\frac{\partial Q_{ph}(r,z,t)}{\partial t}=-L\frac{df_{L}(T)}{dT}\frac{\partial T(r,z,t)}{\partial t}-\\ \tau_{q}L\left\{\frac{d^{2}f_{L}(T)}{dT^{2}}{\left[\frac{\partial T(r,z,t)}{\partial t}\right]}^{2}+\frac{df_{L}(T)}{dT}\frac{\partial^{2}T(r,z,t)}{\partial t^{2}}\right\} \end{array}.$$

Very often the course of the function *f_S_* between the border temperatures is assumed in the linear form, e.g., [46]

(12) $$f_{L}(r,z,t)=\frac{T(r,z,t)-T_{1}}{T_{2}-T_{1}}.$$

One can see that this function fulfils the conditions *f_L_* (*T*_1_) = 0 and *f_L_* (*T*_2_) = 1. The first derivative of this function is equal to 1/(*T*_2_ − *T*_1_), while the second derivative is equal to 0 and the source function (11) takes a form

(13) $$\begin{array}{l} Q_{ph}(r,z,t)+\tau_{q}\frac{\partial Q_{ph}(r,z,t)}{\partial t}=-L\frac{df_{L}(T)}{dT}\left[\frac{\partial T(r,z,t)}{\partial t}+\tau_{q}\frac{\partial^{2}T(r,z,t)}{\partial t^{2}}\right]=\\ -\frac{L}{T_{2}-T_{1}}\left[\frac{\partial T(r,z,t)}{\partial t}+\tau_{q}\frac{\partial^{2}T(r,z,t)}{\partial t^{2}}\right] \end{array}.$$

Thus, the Equation (7) can be written as follows

(14) $$\begin{array}{l} C(T)\left[\frac{\partial T(r,z,t)}{\partial t}+\tau_{q}\frac{\partial^{2}T(r,z,t)}{\partial t^{2}}\right]=\nabla \left[\lambda (T)\nabla T(r,z,t)\right]+\\ \tau_{T}\frac{\partial }{\partial t}\left\{\nabla \left[\lambda (T)\nabla T(r,z,t)\right]\right\}+Q_{L}(r,z,t)+\tau_{q}\frac{\partial Q_{L}(r,z,t)}{\partial t} \end{array},$$

while the parameter *C* is called ‘a substitute thermal capacity’ and

(15) $$C(T)=\left\{ \begin{array}{cc} c_{L} & T>T_{2}\\ c_{M}+\frac{L}{T_{2}-T_{1}} & T_{1}\leq T\leq T_{2}\\ c_{S} & T<T_{1} \end{array}\right.,$$

where $c_{L}$, $c_{S}$ are the volumetric specific heats of liquid and solid state, while (for example) $c_{M}$ is the arithmetic mean of $c_{L}$ and $c_{S}$. the dual phase la.

Additionally, the following definition of the thermal conductivity is introduced

(16) $$\lambda (T)=\left\{ \begin{array}{cc} \lambda_{L} & T>T_{2}\\ \lambda_{M} & T_{1}\leq T\leq T_{2}\\ \lambda_{S} & T<T_{1} \end{array}\right.,$$

where λ*_L_*, λ*_S_* are the thermal conductivities of liquid and solid state, while (for example) λ*_M_* is the arithmetic mean of λ*_L_* and λ*_S_*.

It should be pointed out that Equation (14) containing the parameters *C*(*T*) and λ(*T*) defined according to (15) and (16) describes the thermal processes proceeding in a whole, conventionally homogeneous metal domain. In the case of the macroscopic melting/solidification models such an approach can be called ‘a one-domain method’.

The mushy zone sub-domain limited by the border temperatures *T*_1_ and *T*_2_ appears in the case of the typical alloys melting/solidification. The melting or solidification of the pure metals proceeds at the constant temperature T*. To apply the one-domain approach for this case, the artificial mushy zone should be introduced. The artificial mushy zone is generated by ‘a stretching’ of solidification point T* to a certain interval [T* − ∆*T*, T* + ∆*T*] and for this interval the function *f_L_* is assumed in the linear form (as previously), this means

(17) $$f_{L}(r,z,t)=\frac{T(r,z,t)-T^{*}+\Delta T}{2\Delta T},$$

and then

(18) $$C(T)=\left\{ \begin{array}{cc} c_{L} & T>T^{*}+\Delta T\\ c_{M}+\frac{L}{2\Delta T} & T^{*}-\Delta T\leq T\leq T^{*}+\Delta T\\ c_{S} & T<T^{*}-\Delta T \end{array}\right..$$

The testing computations show that the assumed value of the interval ∆*T* (within a reasonable range of the few degrees e.g., 2.5–4.5 K) does not change significantly the results of numerical simulations [47].

## 4. Two Forms of the Dual Phase Lag Equation

The other approach to the numerical modeling of the melting/resolidification process discussed in this paper consists of the local application of two mutually exclusive forms of Equation (7). For the sub-domain in which the temperature did not reach the melting point, the dual phase lag equation with the source function (9) resulting from laser action is used, while the source function related to the melting and resolidification is equal to zero. The same situation takes place at the stage of the cooling process. On the other hand, in the sub-domain where the temperature is equal to the melting point, the source function related to the melting/resolidification process must be taken into account, while the temperature derivatives with respect to time are equal to zero.

Let us rewrite the Equation (7) with more precisely defined source function (9) controlling the melting/resolidification process

(19) $$\begin{array}{l} c\left[\frac{\partial T(r,z,t)}{\partial t}+\tau_{q}\frac{\partial^{2}T(r,z,t)}{\partial t^{2}}\right]=\nabla \left[\lambda \nabla T(r,z,t)\right]+\\ \tau_{T}\frac{\partial }{\partial t}\left\{\nabla \left[\lambda \nabla T(r,z,t)\right]\right\}+Q_{L}(r,z,t)+\tau_{q}\frac{\partial Q_{L}(r,z,t)}{\partial t}-\\ L\frac{\partial f_{L}(r,z,t)}{\partial t}-\tau_{q}L\frac{\partial^{2}f_{L}(r,z,t)}{\partial t^{2}} \end{array}.$$

Parameters *c* and λ differ for solid and liquid phases, of course.

At the stage of cooling/heating process the function *f_L_* takes values 0 or 1 (solid or liquid) and the source function related to the melting/resolidification is equal to zero. Thus, for the subdomains in which the cooling/heating process takes place one obtains

(20) $$\begin{array}{l} c\left[\frac{\partial T(r,z,t)}{\partial t}+\tau_{q}\frac{\partial^{2}T(r,z,t)}{\partial t^{2}}\right]=\nabla \left[\lambda \nabla T(r,z,t)\right]+\\ \tau_{T}\frac{\partial }{\partial t}\left\{\nabla \left[\lambda \nabla T(r,z,t)\right]\right\}+Q_{L}(r,z,t)+\tau_{q}\frac{\partial Q_{L}(r,z,t)}{\partial t} \end{array}.$$

In turn, for the subdomains being at the stage of melting/resolidification process, the temperature is a constant value (corresponding to the solidification point T*) and then the cooling/heating rate is equal to zero, while the Equation (19) is of the form

(21) $$\begin{array}{l} L\left[\frac{\partial f_{L}(r,z,t)}{\partial t}+\tau_{q}\frac{\partial^{2}f_{L}(r,z,t)}{\partial t^{2}}\right]=\nabla \left[\lambda \nabla T(r,z,t)\right]+\\ \tau_{T}\frac{\partial }{\partial t}\left\{\nabla \left[\lambda \nabla T(r,z,t)\right]\right\}+Q_{L}(r,z,t)+\tau_{q}\frac{\partial Q_{L}(r,z,t)}{\partial t} \end{array}.$$

At the stage of numerical computations the implicit scheme of FDM is used. For each grid node the control volume (here in the form of rings) is assigned. Depending on the current situation the thermal processes in the volume considered are described by Equation (20) or (21).

## 5. Boundary and Initial Conditions

The cylindrical domain is limited by the side surface and two bases. The surfaces *r* = *R*_0_ (external radius of domain) and *z* = *Z* (bottom base of cylinder) are far enough away from the source resulting from the laser’s action, that adiabatic conditions can be accepted both for *R*_0_ and *Z*. A similar condition can be assumed for *z* = 0. The laser action is taken into account by the introduction of an internal heat source, while the time of heat released outside (at the second stage of the process) is very short and this effect can be omitted.

The no-flux condition in the case of the first-order DPLE application is of the form [14,30]

(22) $$-\lambda \left[n\cdot \nabla T\left(r,z,t\right)+\tau_{T}\frac{\partial \left[n\cdot \nabla T\left(r,z,t\right)\right]}{\partial t}\right]=0,$$

where **n** is the normal outward vector, n⋅∇T(r,z,t) is the derivative of temperature in the normal direction. In the considered case the directional derivative corresponds to ∂*T*/∂*r* or ± ∂*T*/∂*z*.

The initial temperature of the metal domain and the initial heating rate are also known

(23) $$t=0:T(r,z,0)=T_{p},\;{\frac{\partial T(r,z,t)}{\partial t}|}_{t=0}=\frac{Q_{L}(r,z,0)}{c},$$

where *T_p_* is the constant initial temperature.

## 6. Numerical Algorithm

To solve the problem discussed, the implicit scheme of the FDM is used. At first, the uniform time grid with the constant time step Δ*t* is introduced

(24) $$0=t^{0}<t^{1}<\ldots <t^{f-2}<t^{f-1}<t^{f}<\ldots <t^{F}<\infty .$$

The axially symmetrical metal domain with dimensions *R*_0_ and *Z* is covered by the regular geometrical mesh with step *h*, as shown in Figure 1. The temperature $T\left(r_{i},z_{j},t^{f}\right)$ for time *t^f^* = *f*·Δ*t* (*f* ≥ 2) at the node (*i*, *j*) is denoted as $T_{i,j}^{f}.$

The FDM algorithm will be described for the heating/cooling processes. The FDM equations for the melting/resolidification stages are very similar.

The following finite difference approximation of Equation (20) is proposed

(25) $$\begin{array}{l} c_{i,j}^{f}\left[\frac{T_{i,j}^{f}-T_{i,j}^{f-1}}{\Delta t}+\tau_{q}\frac{T_{i,j}^{f}-2T_{i,j}^{f-1}+T_{i,j}^{f-2}}{{\left(\Delta t\right)}^{2}}\right]=\frac{\Delta t+\tau_{T}}{\Delta t}\nabla {\left[\lambda \nabla T(r,z,t)\right]}_{i,j}^{f}-\\ \frac{\tau_{T}}{\Delta t}\nabla {\left[\lambda \nabla T(r,z,t)\right]}_{i,j}^{f-1}+{\left(Q_{L}\right)}_{i,j}^{f}+\tau_{q}{\left(\frac{\partial Q_{L}}{\partial t}\right)}_{i,j}^{f} \end{array},$$

where (c.f. [32,45])

(26) $${\left[\nabla \left(\lambda \nabla T\right)\right]}_{i,j}^{s}=\Phi_{i,j1}\frac{T_{i,j-1}^{s}-T_{i,j}^{s}}{R_{i,j1}^{s}}+\Phi_{i,j2}\frac{T_{i,j+1}^{s}-T_{i,j}^{s}}{R_{i,j2}^{s}}+\Phi_{i,j3}\frac{T_{i-1,j}^{s}-T_{i,j}^{s}}{R_{i,j3}^{s}}+\Phi_{i,j4}\frac{T_{i+1,j}^{s}-T_{i,j}^{s}}{R_{i,j4}^{s}}.$$

In the case of the axially symmetrical domain

(27) $$\begin{array}{ccc} \Phi_{i,j1}=\frac{r_{i,j}-0.5h}{hr_{i,j}}, & \Phi_{i,j2}=\frac{r_{i,j}+0.5h}{hr_{i,j}}, & \Phi_{3}=\Phi_{4}=\frac{1}{h} \end{array},$$

and

(28) $$\begin{array}{ll} R_{i,j1}^{s}=\frac{0.5h}{\lambda_{i,j}^{s}}+\frac{0.5h}{\lambda_{i,j-1}^{s}}, & R_{i,j2}^{s}=\frac{0.5h}{\lambda_{i,j}^{s}}+\frac{0.5}{\lambda_{i,j+1}^{s}},\\ R_{i,j3}^{s}=\frac{0.5h}{\lambda_{i,j}^{s}}+\frac{0.5h}{\lambda_{i-1,j}^{s}}, & R_{i,j4}^{s}=\frac{0.5h}{\lambda_{i,j}^{s}}+\frac{0.5h}{\lambda_{i+1,j}^{s}} \end{array}.$$

The functions Φ can be called the shape functions of the FDM mesh, while *R* are the thermal resistances between the neighboring nodes, time level *s* = *f* or *s* = *f* − 1. The detailed mathematical considerations concerning the FDM approximation of the operator ∇(λ∇T) for different co-ordinate systems can be found in [45].

Thus, the Equation (25) can be written in the form

(29) $$\begin{array}{l} c_{i,j}^{f}\left[\frac{T_{i,j}^{f}-T_{i,j}^{f-1}}{\Delta t}+\tau_{q}\frac{T_{i,j}^{f}-2T_{i,j}^{f-1}+T_{i,j}^{f-2}}{{\left(\Delta t\right)}^{2}}\right]=\\ \frac{\Delta t+\tau_{T}}{\Delta t}\left(\Phi_{i,j1}\frac{T_{i,j-1}^{f}-T_{i,j}^{f}}{R_{i,j1}^{f}}+\Phi_{i,j2}\frac{T_{i,j+1}^{f}-T_{i,j}^{f}}{R_{i,j2}^{f}}+\Phi_{3}\frac{T_{i-1,j}^{f}-T_{i,j}^{f}}{R_{i,j3}^{f}}+\Phi_{4}\frac{T_{i+1,j}^{f}-T_{i,j}^{f}}{R_{i,j4}^{f}}\right)-\\ \frac{\tau_{T}}{\Delta t}\left(\Phi_{i,j1}\frac{T_{i,j-1}^{f-1}-T_{i,j}^{f-1}}{R_{i,j1}^{f-1}}+\Phi_{i,j2}\frac{T_{i,j+1}^{f-1}-T_{i,j}^{f-1}}{R_{i,j2}^{f-1}}+\Phi_{3}\frac{T_{i-1,j}^{f-1}-T_{i,j}^{f-1}}{R_{i,j3}^{f-1}}+\Phi_{4}\frac{T_{i+1,j}^{f-1}-T_{i,j}^{f-1}}{R_{i,j4}^{f-1}}\right)+\\ {\left(Q_{L}\right)}_{i,j}^{f}+\tau_{q}{\left(\frac{\partial Q_{L}}{\partial t}\right)}_{i,j}^{f} \end{array},$$

or

(30) $$\begin{array}{l} \left[\frac{c_{i,j}^{f}\left(\Delta t+\tau_{q}\right)}{{\left(\Delta t\right)}^{2}}+\frac{\Delta t+\tau_{T}}{\Delta t}\left(\frac{\Phi_{i,j1}}{R_{i,j1}^{f}}+\frac{\Phi_{i,j2}}{R_{i,j2}^{f}}+\frac{\Phi_{3}}{R_{i,j3}^{f}}+\frac{\Phi_{4}}{R_{i,j4}^{f}}\right)\right]T_{i,j}^{f}=\\ \frac{\Delta t+\tau_{T}}{\Delta t}\left(\frac{\Phi_{i,j1}}{R_{i,j1}^{f}}T_{i,j-1}^{f}+\frac{\Phi_{i,j2}}{R_{i,j2}^{f}}T_{i,j+1}^{f}+\frac{\Phi_{3}}{R_{i,j3}^{f}}T_{i-1,j}^{f}+\frac{\Phi_{4}}{R_{i,j4}^{f}}T_{i+1,j}^{f}\right)-\\ \frac{\tau_{T}}{\Delta t}\left(\Phi_{i,j1}\frac{T_{i,j-1}^{f-1}-T_{i,j}^{f-1}}{R_{i,j1}^{f-1}}+\Phi_{i,j2}\frac{T_{i,j+1}^{f-1}-T_{i,j}^{f-1}}{R_{i,j2}^{f-1}}+\Phi_{3}\frac{T_{i-1,j}^{f-1}-T_{i,j}^{f-1}}{R_{i,j3}^{f-1}}+\Phi_{4}\frac{T_{i+1,j}^{f-1}-T_{i,j}^{f-1}}{R_{i,j4}^{f-1}}\right)+\\ \frac{c_{i,j}^{f}(\Delta t+2\tau_{q})}{{\left(\Delta t\right)}^{2}}T_{i,j}^{f-1}-\frac{c_{i,j}^{f}\tau_{q}}{{\left(\Delta t\right)}^{2}}T_{i,j}^{f-2}+{\left(Q_{L}\right)}_{i,j}^{f}+\tau_{q}{\left(\frac{\partial Q_{L}}{\partial t}\right)}_{i,j}^{f} \end{array}.$$

Denoting

(31) $$\begin{array}{l} A_{1}^{f}=\frac{\Delta t+\tau_{T}}{\Delta t}\frac{\Phi_{i,j1}}{R_{i,j1}^{f}},\;A_{2}^{f}=\frac{\Delta t+\tau_{T}}{\Delta t}\frac{\Phi_{i,j2}}{R_{i,j2}^{f}},\;A_{3}^{f}=\frac{\Delta t+\tau_{T}}{\Delta t}\frac{\Phi_{3}}{R_{i,j3}^{f}},\\ A_{4}^{f}=\frac{\Delta t+\tau_{T}}{\Delta t}\frac{\Phi_{4}}{R_{i,j4}^{f}},\;A_{0}^{f}=A_{1}^{f}+A_{2}^{f}+A_{3}^{f}+A_{4}^{f}+\frac{c_{i,j}^{f}\left(\Delta t+\tau_{q}\right)}{{\left(\Delta t\right)}^{2}} \end{array},$$

one obtains

(32) $$\begin{array}{l} T_{i,j}^{f}=\frac{1}{A_{0}^{f}}\left(A_{1}^{f}T_{i,j-1}^{f}+A_{2}^{f}T_{i,j+1}^{f}+A_{3}^{f}T_{i-1,j}^{f}+A_{4}^{f}T_{i+1,j}^{f}\right)-\\ \frac{\tau_{T}}{A_{0}^{f}\Delta t}\left(\Phi_{i,j1}\frac{T_{i,j-1}^{f-1}-T_{i,j}^{f-1}}{R_{i,j1}^{f-1}}+\Phi_{i,j2}\frac{T_{i,j+1}^{f-1}-T_{i,j}^{f-1}}{R_{i,j2}^{f-1}}+\Phi_{3}\frac{T_{i-1,j}^{f-1}-T_{i,j}^{f-1}}{R_{i,j3}^{f-1}}+\Phi_{4}\frac{T_{i+1,j}^{f-1}-T_{i,j}^{f-1}}{R_{i,j4}^{f-1}}\right)+\\ \frac{c_{i,j}^{f}(\Delta t+2\tau_{q})}{A_{0}^{f}{\left(\Delta t\right)}^{2}}T_{i,j}^{f-1}-\frac{c_{i,j}^{f}\tau_{q}}{A_{0}^{f}{\left(\Delta t\right)}^{2}}T_{i,j}^{f-2}+\frac{1}{A_{0}^{f}}\left[{\left(Q_{L}\right)}_{i,j}^{f}+\tau_{q}{\left(\frac{\partial Q_{L}}{\partial t}\right)}_{i,j}^{f}\right] \end{array}.$$

The approximation of the no-flux boundary conditions (22) is assumed in the form

(33) $$\frac{\Delta t+\tau_{T}}{\Delta t}{\left[n\cdot \nabla T\left(r,z,t\right)\right]}^{f}-\frac{\tau_{T}}{\Delta t}{\left[n\cdot \nabla T\left(r,z,t\right)\right]}^{f-1}=0.$$

Because the normal derivative for the bottom and upper surface is equal to ± ∂*T*/∂*z*, while for the cylinder side ∂*T*/∂*r*, consequently the approximation of n⋅∇T(r,z,t) is very simple (the left and right sides differential quotients are used). Thus, the final form of conditions (33) will not be presented here. 

Obtained in this way the system of linear equations for transition *t^f^* ^− 1^ → *t^f^* is solved using the iterative method. It should be noted that the presented algorithm is unconditionally stable [34].

## 7. Results of Computations

The calculations presented below are performed for a thin metal film of dimensions *Z* = 100 nm, *R*_0_ = 100 nm made of chromium and subjected to a laser pulse (laser beam radius *r_D_* = *R*_0_/8, reflectivity *R* = 0.93, optical penetration depth δ = 15.3 nm, c.f. Equation (8)). The mathematical form of internal heat source resulting from the laser action suggests the orientation of the domain in the cylindrical coordinates (axially-symmetrical task). The dimensions *Z* and *R*_0_ of the cylinder conventionally cut from the metal domain are assumed in such a way that the adiabatic conditions on the side and bottom surfaces can be accepted. The following values of chromium thermophysical parameters are assumed: thermal conductivities λ*_S_* = 93 W/(m K), λ*_L_* = 35 W/(m K), volumetric specific heats $c_{S}$ = 3.2148 MJ/(m^3^ K), $c_{L}$ = 2.79276 MJ/(m^3^ K) melting point T* = 2180 K, volumetric heat of fusion *L* = 2904 MJ/m^3^ [48], relaxation time $\tau_{q}$ = 0.136 ps, thermalization time $\tau_{T}$ = 7.86 ps [1]. The solution obtained for the artificial mushy zone model corresponds to ∆*T* = 3 K, while the thermal conductivity and volumetric specific heat of this sub-domain are equal to the arithmetic means of the liquid and solid parameters. The initial temperature of the domain is equal to *T_p_* = 300 K. The step of the geometrical regular mesh *h* = 2 nm, while the time step ∆*t* = 0.0002 ps (the testing computations show that the further compaction of the grid does not change the numerical results).

The main purpose of the computations is to compare the melting/resolidification modeling results using two models, namely: model 1—melting at the temperature interval (artificial mushy zone), model 2—melting at the constant temperature.

The laser intensity and the characteristic time of the laser pulse are selected in such a way that the melting and resolidification take place, but the evaporation process does not appear. At the beginning, the laser intensity *I*_0_ = 1.6 × 10^4^ J/m^2^ and three values of the characteristic times of laser pulse *t_p_* = 5 ps, *t_p_* = 10 ps, *t_p_* = 11 ps are taken into account (for *t_p_* > 11 ps the phase change has no place).

**Figure 2:**
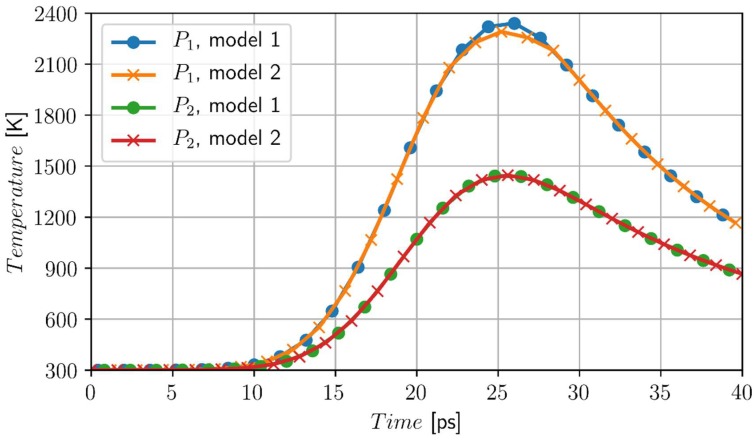
Temperature history at the points *P*_1_ (0, 0) and *P*_2_ (0, 20 nm), model 1—artificial mushy zone (∆*T* = 3 K), model 2—melting at the constant temperature, chromium, *I*_0_ = 1.6 × 10^4^ J/m^2^, *t_p_* = 10 ps.

**Figure 3:**
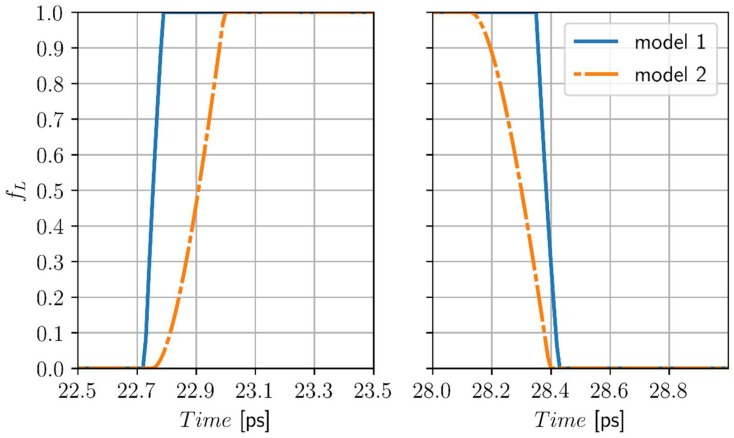
Changes of the volumetric liquid state at the point *P*_1_ (0, 0), model 1—artificial mushy zone (∆*T* = 3 K), model 2—melting at the constant temperature, chromium, *I*_0_ = 1.6 × 10^4^ J/m^2^, *t_p_* = 10 ps.

In Figure 2, the heating/cooling curves at points *P*_1_ (0, 0) and *P*_2_ (0, 20 nm) for *t_p_* = 10 ps are shown. At the most heated point *P*_1_ the differences are visible, but at the other points (nodes) the courses of heating and cooling curves are almost identical. The next Figure shows the changes of the volumetric liquid state fraction at the point *P*_1_. The left part of Figure 3 illustrates the melting process, while the right part of this figure illustrates the resolidification process. Here, the differences are more visible, although the durations of these processes are comparable. The linear course of *f_L_* corresponds to the artificial mushy zone model (it results from theoretical considerations—Equation (12)).

**Figure 4:**
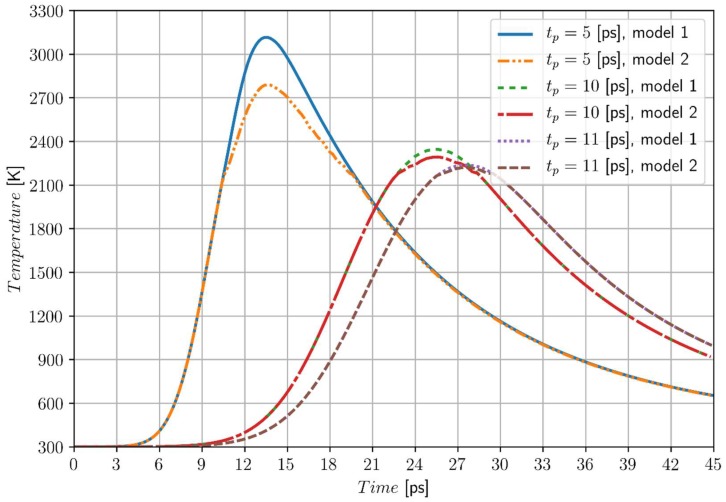
Temperature history at the point *P*_1_ (0, 0), model 1—artificial mushy zone (∆*T* = 3 K), model 2—melting at the constant temperature, chromium, *I*_0_ = 1.6 × 10^4^ J/m^2.^

In Figure 4, the temperature histories at point *P*_1_ (0, 0) for different values of characteristic times of laser pulse (for both models) are shown. Analysis of the mathematical form of the source function (8) confirms that for a smaller value of *t_p_*, the value of *Q_L_* (*r*, *z*, *t*) is greater, which causes that a higher local maximum of temperature is achieved. One can see that the time after which the local temperature reaches the maximum is significantly shorter.

The comparison of the obtained solutions shows, that the maximum differences between the temperatures determined by two considered models are equal to 325.5 K for *t_p_* = 5 ps, 51.5 K for *t_p_* = 10 ps and 17.1 K for *t_p_* = 11 ps. Smaller differences are observed for higher values of *t_p_*.

**Figure 5:**
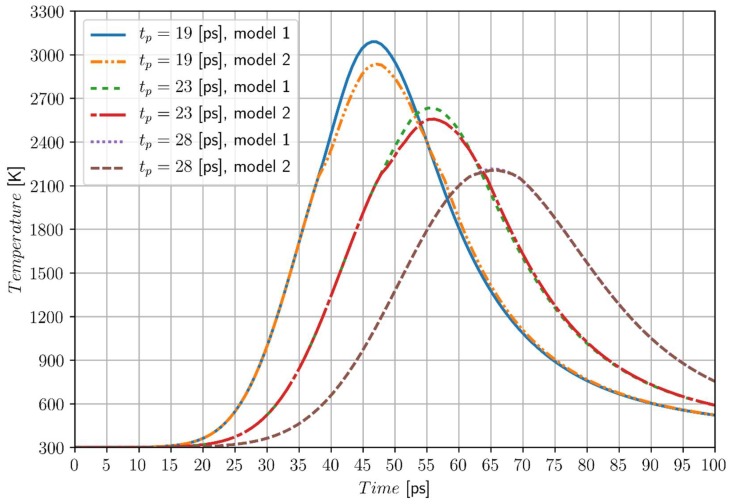
Temperature history at the point *P*_1_ (0, 0), model 1—artificial mushy zone (∆*T* =3 K), model 2—melting at the constant temperature, chromium, *I*_0_ = 2.6 × 10^4^ J/m^2.^

Similar computations are performed for the greater value of laser intensity, namely *I*_0_ = 2.6 × 10^4^ J/m^2^ and characteristic times of laser pulse *t_p_* = 19 ps, *t_p_* = 23 ps and *t_p_* = 28 ps, respectively (for *t_p_* < 19 ps the evaporation temperature is reached, while for *t_p_* > 28 ps the phase change has no place). In Figure 5 the temperature histories at the point *P*_1_ (0, 0) for different values of characteristic time of laser pulse and for both models are shown. The following differences are obtained: 149.2 K for *t_p_* = 19 ps, 75.7 K for *t_p_* = 23 ps and 8.3 K for *t_p_* = 28 ps.

Comparing the two solutions presented above (Figure 4 and Figure 5), one can notice that for the greater laser intensities and the greater characteristic times of laser pulse, the solution resulting from the artificial mushy zone model is closer to the results corresponding to the two forms of DPLE application.

**Figure 6:**
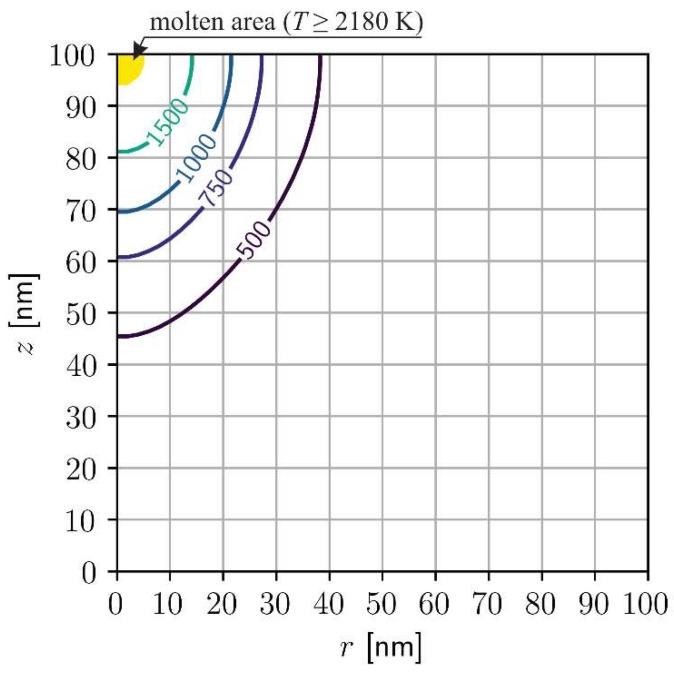
Temperature distribution after 25 ps, melting at the constant temperature, chromium, *I*_0_ = 1.6 × 10^4^ J/m^2^, *t_p_* = 10 ps.

The results of numerical computations allow, among others, to observe the temporary shape and dimensions of the pool of molten metal. In Figure 6 the final shape of the pool before the stage of resolidification is presented.

To investigate more accurately the ‘action’ of the model using two forms of DPLE, the material with significantly different (in comparison with chromium) thermophysical parameters is taken into account. Thus, the cylindrical domain (*R*_0_ = 100 nm, *Z* = 100 nm) made of gold is considered. The material parameters are the following: λ*_S_* = 315 W/(m K), λ*_L_* = 105 W/(m K), volumetric specific heats $c_{S}$ = 2.4897 MJ/(m^3^ K), $c_{L}$ = 2.8757 MJ/(m^3^ K) melting temperature T* =1336 K, volumetric heat of fusion *L* = 1229.99 MJ/m^3^. Relaxation time $\tau_{q}$ = 8.5 ps, thermalization time $\tau_{T}$ = 90 ps [49]. The laser beam parameters: $r_{D}$ = *R*_0_/8, laser intensity: *I*_0_ = 5 × 10^4^ J/m^2^, characteristic time of laser pulse: *t_p_* = 5 ps. The initial temperature of domain is equal to *T_p_* = 300 K.

**Figure 7:**
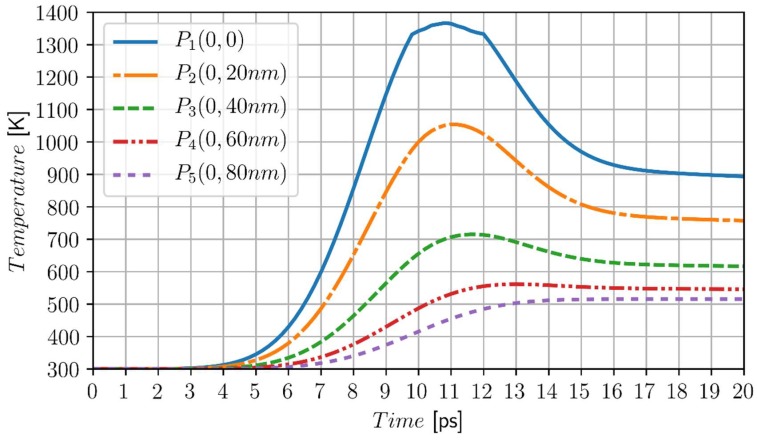
Temperature history at the points *P*_1_ (0, 0), *P*_2_ (0, 20 nm), *P*_3_(0, 40 nm), *P*_4_ (0, 60 nm) and *P*_5_(0, 80 nm), gold, *I*_0_ = 5 × 10^4^ J/m^2^, *t_p_* = 5 ps.

In Figure 7, the heating/cooling curves at the selected points from the metal domain are shown.

The results of computations are (from a qualitative point of view) similar to those obtained previously. It can be seen that the maximum temperature at the node (0, 0) is obtained after the time of 11 ps, while in the case of chromium, this time is equal to 13.5 ps (for the same characteristic time of laser pulse). This may be surprising because chromium should be heated to a much higher temperature to initiate the melting process, which should last longer. It results from the fact that the thermal conductivity of the gold is significantly bigger, which causes the some equalization of the temperature field in the domain and significantly reduces local temperature gradients (heat fluxes). Additionally, the lag times of gold are much longer than in the case of chromium. These factors mean that despite such different materials, the heating/cooling curves with respect to time are quite similar. The local and temporary temperatures are, of course, quite different.

## 8. Conclusions

Two variants of numerical modeling of melting and resolidification occurring in thin metal films subjected to an ultrashort laser pulse have been discussed. In the first variant, the artificial mushy zone was introduced (melting and resolidification in the interval temperature), while the second variant was based on two forms of a dual phase lag equation. From the numerical point of view, the developed computer programs are of similar complexity, although the program for the artificial mushy zone approach is somewhat simpler.

The discussion of the obtained results has been presented in detail in Section 7. Summing up, in the case of microscale heat transfer modeling the numerical solutions based on the two proposed models to some extent differ from each other, but it seems that in the practical applications such differences are acceptable. The closer results of solutions have been obtained for the greater values of characteristic times of laser pulse *t_p_*. The heating/cooling curves presented in the previous section concerned the most intense regions of the heat exchange, while in the peripheral sub-domains, the differences between the solutions were insignificant.

The authors’ approach to phase change modeling was not used. Taking into account the micro-technologies based on the melting or solidification phenomena, one can believe that this type of numerical models should be useful at the stage of technology design.

Further research in this area is expectd to tackle the phenomenon of ablation caused by the heating of material to very high temperatures.

## Definition of Symbols

The following symbols are used in this manuscript:


*c*
volumetric specific heat [W/(m^3^K)]
*C*
substitute thermal capacity [W/(m^3^K)]
*f*
level of time
*f_S_*
volumetric solid-state fraction
*f_L_*
volumetric liquid state fraction
*I*_0_
laser intensity [J/m^2^]
*L*
volumetric heat of fusion [J/m^3^]
**q**
heat flux vector [W/m^2^]
*Q*
capacity of internal heat sources [W/m^3^]
*Q_L_*
source function resulting from the laser action [W/m^3^]
*Q_ph_*
source function related to melting [W/m^3^]
*R*
reflectivity of the irradiated surface
*R*_0_
domain radius [m]
*r_D_*
laser beam radius [m]
*T*
temperature [K]*T* *melting temperature [K][*T*_1_, *T*_2_]temperature interval in which the melting process takes place
*T_p_*
initial temperature [K]
*t*
time [s]
*t_p_*
characteristic time of laser pulse [s]*X* = {*r*, *z*}geometrical co-ordinates
*Z*
domain depth [m]Greeks:δoptical penetration depth [m]λthermal conductivity [W/(mK)]τ*_T_*thermalization time [s]τ*_q_*relaxation time [s]
